# Targeting HPV‐infected cervical cancer cells with PEGylated liposomes encapsulating siRNA and the role of siRNA complexation with polyethylenimine

**DOI:** 10.1002/btm2.10022

**Published:** 2016-08-08

**Authors:** Rachel M. Levine, Christina V. Dinh, Michael A. Harris, Efrosini Kokkoli

**Affiliations:** ^1^ Dept. of Chemical Engineering and Materials Science University of Minnesota Minneapolis MN 55455

**Keywords:** si18E7‐674 siRNA, gene delivery, stealth liposomes, targeted delivery, AG86 peptide, alpha6beta4 integrin, peptide‐amphiphiles, cervical cancer

## Abstract

The greatest obstacle to clinical application of cancer gene therapy is lack of effective delivery tools. Gene delivery vehicles must protect against degradation, avoid immunogenic effects and prevent off target delivery which can cause harmful side effects. PEGylated liposomes have greatly improved tumor localization of small molecule drugs and are a promising tool for nucleic acid delivery as the polyethylene glycol (PEG) coating protects against immune recognition and blood clearance. In this study, small interfering RNA (siRNA) was fully encapsulated within PEGylated liposomes by complexing the siRNA with a cationic polymer, polyethyleneimine (PEI), before encapsulation. Formation methods and material compositions were then investigated for their effects on encapsulation. This technology was translated for protective delivery of siRNA designed for human papillomavirus (HPV) viral gene silencing and cervical cancer treatment. PEGylated liposomes encapsulating siRNA were functionalized with the AG86 targeting peptide‐amphiphile which binds to the α_6_β_4_ integrin, a cervical cancer biomarker. It was found that both targeting and polymer complexation before encapsulation were critical components to effective transfection.

## 1 Introduction

Rapid advancements in genetic technologies have given researchers the ability to target and modify individual genetic events involved in disease progression, providing potential treatment avenues for previously untreatable diseases.^1^, ^2^, ^3^ A particularly valuable tool for characterization and modification of disease‐associated genes is RNA interference (RNAi), a cellular pathway which can selectively silence the expression of a target gene. When short (∼22 base pair) RNA sequences are introduced into the cytoplasm of a cell, they can be incorporated into an RNA‐induced silencing complex. The RNA‐induced silencing complex is molecular machinery which identifies the complementary mRNA sequence of the incorporated RNA for degradation, thus preventing translation and expression of the target gene. Selective gene silencing can be especially useful for treating diseases such as cancer or viral infection, where disease progression is driven by undesirable or aberrant gene expression.^2^, ^4^, ^5^ One such example of the potential of RNAi therapy is demonstrated by the treatment of cervical cancer by silencing key genes within the cellularly integrated genome of the oncovirus HPV. The oncogenic nature of HPV has been attributed to the aberrant expression of the E6 and E7 viral proteins, and their interference with native cell cycle regulatory pathways. E6 and E7 bind to tumor suppressor proteins p53 and pRb, marking them for degradation or blocking their binding sites, thereby preventing apoptosis and driving cellular proliferation.^6^, ^7^ With the knowledge of the genetic mechanism of this oncovirus, several siRNA sequences targeting the gene sequences that encode the E6 and E7 proteins have been developed, demonstrating rescue of the p53 and pRb tumor suppression pathways, resulting in cell cycle arrest and apoptosis in HPV‐infected cancer cells both *in vitro* and *in vivo*.^8^, ^9^, ^10^, ^11^, ^12^

**Figure 1:**
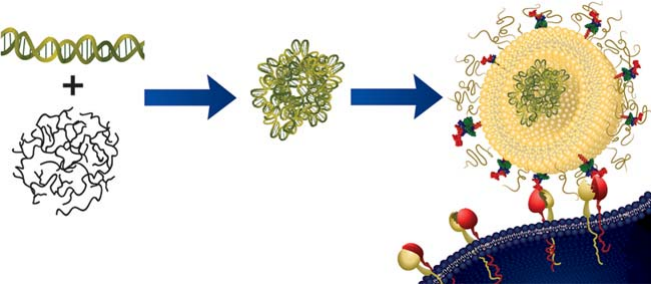
Targeted PEGylated liposomes encapsulating siRNA/PEI complexes. si18E7‐674 shown to silence the HPV‐E7 gene was complexed with PEI, encapsulated into AG86‐functionalized PEGylated liposomes and delivered to α_6_β_4_‐expressing HPV‐18 containing HeLa cervical cancer cells

In order for the potential of clinical gene therapy to be realized, several key obstacles to efficient *in vivo* delivery need to be overcome. For successful transfection and therapy to occur, siRNA must be internalized into the cells and released into the cytosol to mediate gene silencing. While traversing the blood stream to reach the target tissue, siRNA must avoid degradation by nucleases, recognition by the immune system, and renal clearance. Several technologies have been developed to address each of these barriers to siRNA delivery, including chemical modification, nanoparticle complexation, and addition of targeting moieties.^1^, ^2^, ^3^, ^4^, ^5^, ^13^ In this study, we developed cancer gene therapy delivery vehicles composed of targeted PEGylated liposomes encapsulating siRNA. PEGylated liposomes are hollow, spherical phospholipid nanoparticles functionalized with a layer of PEG which have seen clinical success for the intravenous (IV) delivery of chemotherapeutic agents.^14^, ^15^, ^16^ PEGylation has been shown to increase blood circulation, minimize immunogenicity and increase tumor accumulation of IV delivered liposomes.^14^, ^15^, ^16^ PEGylated liposomes present a potential solution to the toxicities observed from traditional cationic siRNA transfection agents such as PEI and 1,2‐dioleoyl‐3‐trimethylammonium‐propane.^17^, ^18^, ^19^ Liposomes functionalized with ligands designed to bind to upregulated surface receptors can enhance cellular association and internalization into cancer cells. The α_6_β_4_ integrins are upregulated surface receptors associated with metastatic behavior in several cancer types, including cervical cancer.^20^, ^21^, ^22^ The AG86 peptide was identified as an α_6_ integrin binding ligand,^23^ and was investigated here for specificity for the α_6_β_4_ integrin and for targeting to HeLa cervical cancer cells. PEI complexation with nucleic acids alone has been shown to aid in endosomal escape through the proton sponge effect, whereby the high buffering capacity of PEI can cause osmotic swelling and rupture of intracellular organelles.^24^, ^25^, ^26^ Previously, we have demonstrated successful encapsulation of plasmid DNA (pDNA) complexed with PEI within the aqueous core of PEGylated liposomes.^27^ With the addition of a targeting ligand, liposome encapsulated PEI‐complexed DNA achieved efficient transfection in colorectal cancer cells.^28^, ^29^ We therefore hypothesized that siRNA/PEI complexation could enhance transfection efficiency within PEGylated liposomes. We engineered AG86‐functionalized PEGylated liposomes encapsulating PEI complexed siRNA (Figure 1) as a delivery scheme to address each of the barriers to effective IV gene delivery. Optimal targeting and complexation properties of this vehicle were identified for successful gene silencing of the HPV‐E7 gene in cervical cancer cells.

## 2 Results

### 2.1 Binding and internalization of fluorescent liposomes delivered to HeLa cells

**Figure 2:**
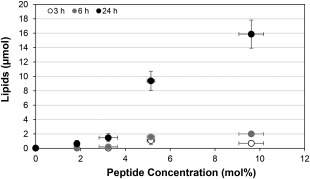
Binding and internalization of fluorescent targeted PEGylated liposomes. 0–10 mol% AG86‐functionalized, calcein loaded, PEGylated liposomes were delivered at 100 μM lipids to HeLa cells for 3, 6, and 24 hr at 37  °C and binding and internalization was examined by lysing cells and measuring fluorescence. Data are presented as the mean ± SE (*n* = 3, performed in quadruplicate). All *p*‐values from statistical analysis are listed in Supporting Information Table S1

To investigate the ability of AG86‐functionalized PEGylated liposomes to target the α_6_ integrin, calcein loaded liposomes were prepared with 0‐10 mol% AG86 targeting peptide and delivered to HeLa cells for 3, 6 and 24 hr at 37 °C. As delivery time increased, binding and internalization increased for all targeting peptide concentrations. Increasing the concentration of the targeting peptide results in a nonlinear increase in binding and internalization (Figure 2), which is likely mediated by increased peptide valency resulting in binding avidity.^30^, ^31^ Liposomes prepared with 5 mol% AG86 achieved significantly more efficient delivery compared to 0, 2, and 3 mol% peptide at all times (Supporting Information Table S1). Although liposomes functionalized with 9 mol% peptide achieved the highest level of binding, more material is required for their production. 5 mol% peptide was therefore chosen as a sufficient targeting peptide concentration for subsequent gene delivery studies. To further verify that the AG86 peptide was responsible for liposome binding to HeLa cells, the binding of AG86‐functionalized PEGylated liposomes was measured after incubation with free AG86 peptide and compared to binding without peptide blocking (Supporting Information Figure S1A). The presence of the free peptide decreased liposome binding by 98%, confirming AG86‐mediated binding. The initial discovery of the AG86 peptide demonstrated specific interaction with the α_6_ integrin.^23^ The presence of α_6_ antibodies disrupted 40% of cellular adhesion to AG86 peptide coated surfaces.^23^ The α_6_ integrin is known to dimerize with either the β_1_ or the β_4_ integrin,^32^ and while blocking with a β_1_ specific antibody demonstrated that AG86 binding is not specific for the α_6_β_1_ heterodimer, binding to the β_4_ integrin was not explored.^23^ We therefore investigated the binding interactions of the AG86 peptide using antibody blocking of the AG86‐functionalized PEGylated liposomes to cells (Supporting Information Figure S1B). In the presence of anti‐α_6_ and anti‐β_4_ integrin antibodies, liposome binding is decreased by 66% and 86%, respectively, verifying the α_6_β_4_ integrin as the binding target of the AG86 peptide.

### 2.2 Characterization of siRNA/PEI complexes

**Figure 3:**
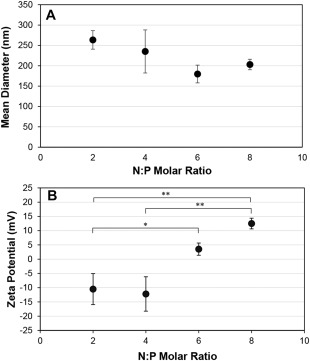
Size (A) and zeta potential (B) measurements of siRNA/PEI complexes. siRNA/PEI particles were complexed at various N:P ratios, and size and charge were determined. Data are presented as the mean ± SE (*n* = 4–11). * *p* < 0.01, ** *p* < 0.001 comparing zeta potential measurements. There was no significant statistical difference for pairs without brackets

Before encapsulation in targeted liposomes, anionic siRNA was complexed with the cationic polymer PEI to form nanoparticles. siRNA was complexed at several different nitrogen:phosphate ratios (N:P) to investigate the effect of N:P ratio on particle size, charge, liposomal encapsulation yield and transfection efficiency. None of the particle sizes are significantly different between different N:P ratios (Figure 3A).^33^ Representative histograms for each N:P ratio are included in Supporting Information Figure S2. As N:P ratio was increased from 2 to 8 and more positively charged polymer was added during complexation, the zeta potential increased from −11 to 13 mV (Figure 3B). The larger standard error of the average particle size for siRNA/PEI complexes at N:P = 4 is indicative of the higher size polydispersity observed from these complexes, as is commonly seen at N:P ratios that produce particles approaching a neutral charge or at the transition from negative to positive zeta potential associated with increasing N:P.^34^, ^35^, ^36^

### 2.3 Isothermal calorimetry (ITC) exploring siRNA/PEI complexation

**Figure 4:**
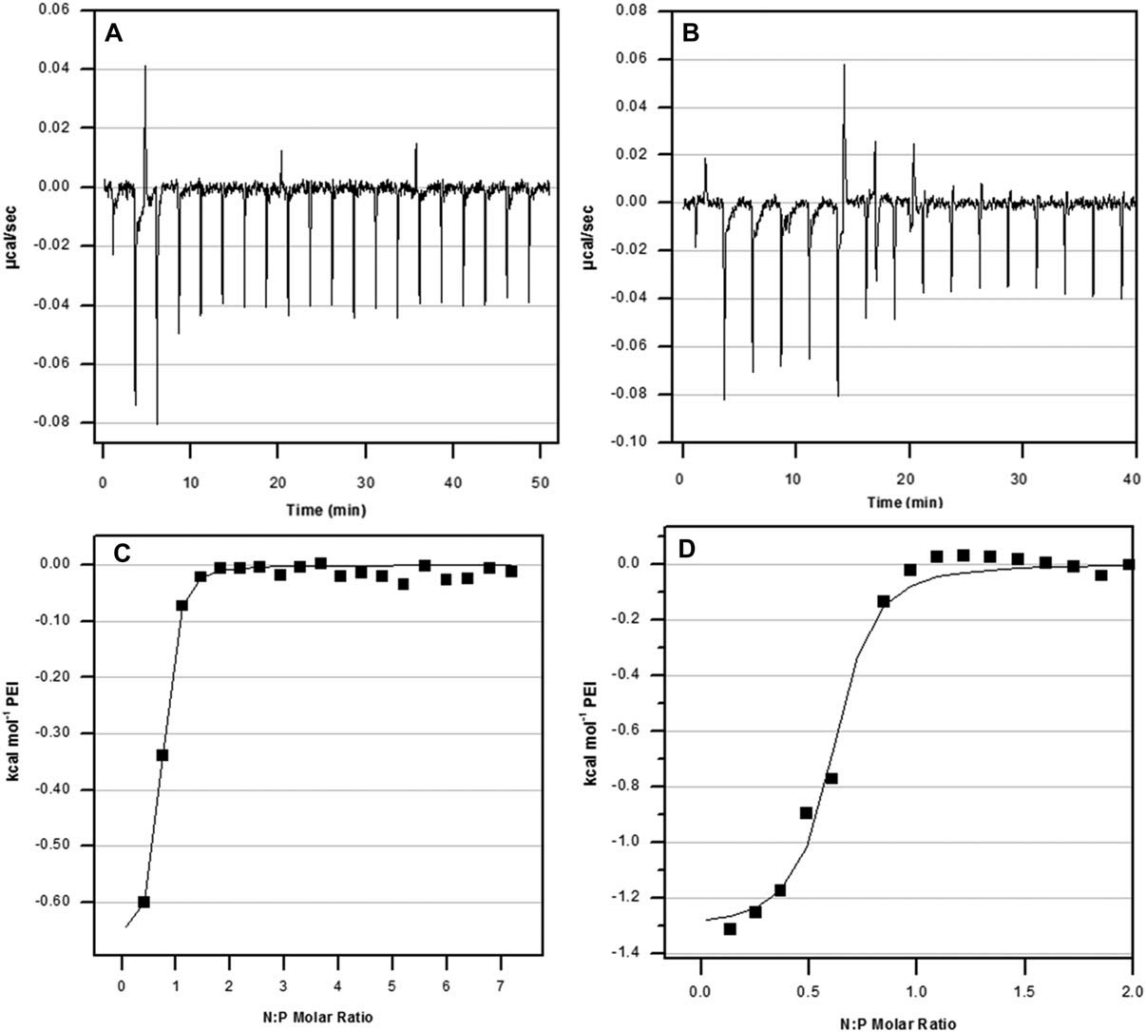
Representative ITC experiments showing raw (A‐B) and integrated (C‐D) data for the titration of PEI into siRNA in 6 mM HEPES buffer. PEI was titrated into siRNA over N:P ranges of 0–8 (A, C) and 0–2 (B, D)

Microcalorimetric titrations of PEI into siRNA solutions were performed to monitor the thermodynamic properties associated with the formation of siRNA/PEI complexes. Titrations were performed over the entire range of N:P ratios investigated for transfection (Figure 4A,C). The calorimetry results over this N:P range showed complete saturation of siRNA with PEI between N:P 1 and 2. Others have observed DNA/PEI saturation between N:P of 2–3 using branched PEI of similar size,^34^, ^37^, ^38^ and between 1 and 2.5 for siRNA/PEI.^39^ In order to better observe the transition from free to fully complexed siRNA, titrations were also performed spanning N:P ratios of 0–2 (Figure 4B,D). A “one set of sites” model was used to calculate binding affinity (*K* = 3.1 × 10^6^ ± 1.2 × 10^6^ M^−1^), enthalpy and entropy of binding (*ΔH* = −1312 ± 50 cal/mol, *ΔS* = 25.3 cal/mol/K), and stoichiometry (*n* = 0.57 ± 0.02). The calculated binding affinity and stoichiometry are similar to those measured from ITC experiments of DNA/PEI complexes.^34^, ^38^ An *n* of 0.57 corresponds to an siRNA:PEI ratio of 25 and a negative to positive charge ratio of 1.7:1. This deviation from a charge ratio of 1:1 could be explained by a difference in linear intercharge spacing between siRNA (0.17 nm)^40^ and PEI (0.25–0.35 nm).^41^ Normalizing to the charge ratio, it is reasonable to expect the interaction of siRNA and PEI to resemble the interaction of DNA with PEI. The binding of siRNA and PEI in distilled water was characterized using similar thermodynamic parameters in the literature, however an *n* of 2.26 was identified, requiring more PEI molecules for condensation of 1 siRNA molecule.^39^ This discrepancy could be caused by differing buffer conditions.^40^ In Figure 4A,B, large endothermic (positive) peaks are observed at the transition between free and complexed siRNA, and this has been attributed to an endothermic reorganization of saturated siRNA:PEI complexes into less siRNA‐dense particles.^39^

### 2.4 Characterization of targeted PEGylated liposomes encapsulating siRNA

**Figure 5:**
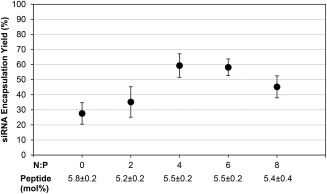
siRNA encapsulation yield in targeted PEGylated liposomes. siRNA/PEI complexes were first prepared at various N:P ratios, then encapsulated in the targeted PEGylated liposomes for characterization. N:P = 0 indicates encapsulation of uncomplexed siRNA (no PEI). Yield is calculated as siRNA present in final liposome solution compared to initial siRNA. Data are presented as the mean ± SE (*n* = 3–8, performed in triplicate). There was no significant statistical difference for all pairs

siRNA/PEI complexes (N:P = 2–8) or uncomplexed siRNA (N:P = 0; no PEI) were encapsulated within 5 mol% AG86‐functionalized PEGylated liposomes. As Supporting Information Figure S3 shows, the N:P ratio had no significant effect on liposome size (average diameters ranged from 116 to 155 nm) or zeta potential (−1.2–8.2 mV) and results were no different than size or zeta potential measured for empty PEGylated liposomes. The yield of siRNA encapsulation was measured using a standard created from the fluorescently labeled siRNA included in the complexes (Figure 5).^27^ Encapsulation of siRNA in neutral lipid delivery systems is historically limited by low entrapment efficiency.^42^ However, targeted PEGylated liposomes encapsulating siRNA complexed to PEI achieved yields of up to 60% as shown in Figure 5.

### 2.5 mRNA silencing from targeted PEGylated liposomes encapsulating siRNA

**Figure 6:**
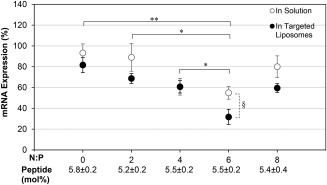
mRNA silencing from siRNA/PEI complexes in solution or encapsulated in targeted PEGylated liposomes. HeLa cells were transfected with 2.5 nM siRNA for 24 hr. mRNA expression was measured as % expression of HPV‐E7 mRNA in HeLa cells compared to untreated cells. N:P = 0 indicates delivery of uncomplexed siRNA (no PEI). Data are presented as the mean ± SE (*n* = 3–7, performed in triplicate). * *p* < 0.01, ** *p* < 0.001 comparing targeted PEGylated liposome‐encapsulated siRNA/PEI, and § *p* < 0.05 comparing targeted PEGylated liposome‐encapsulated siRNA/PEI to siRNA/PEI complexes in solution at the same N:P. There was no significant statistical difference for pairs without brackets

**Figure 7:**
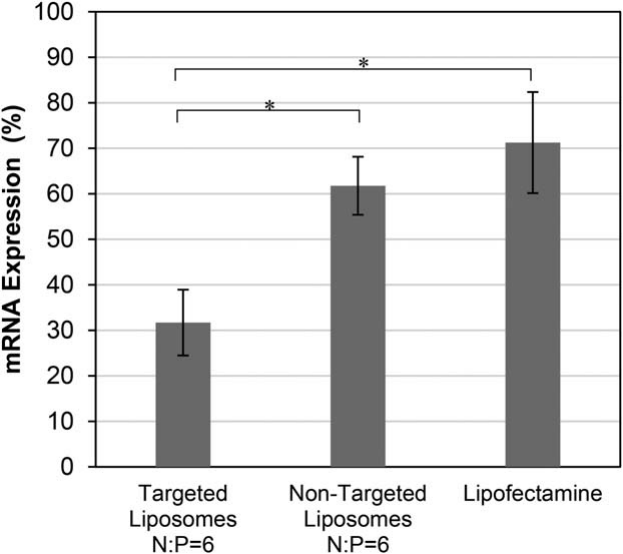
mRNA expression after siRNA transfection with various reagents. siRNA was complexed with PEI at N:P = 6 and encapsulated in targeted (5.5 ± 0.2 mol% AG86) or non‐targeted PEGylated liposomes, or mixed with Lipofectamine. mRNA silencing of HPV‐E7 in HeLa cells was compared to untreated cells, 24 hr after delivery of 2.5 nM of siRNA. Data are presented as the mean ± SE (*n* = 4‐6, performed in triplicate). * *p* < 0.05. There was no significant statistical difference for pairs without brackets

mRNA silencing from the HPV type‐18 specific siRNA sequence^12^ was measured in HPV‐18 containing HeLa cells. siRNA/PEI complexes encapsulated in targeted PEGylated liposomes and free siRNA/PEI were delivered to HeLa cells, and the resulting mRNA silencing from the delivery of HPV‐E7 specific siRNA was measured 24 hr after delivery using qRT‐PCR (Figure 6). The level of mRNA silencing increased with increasing N:P ratio up to N:P = 6 for both the free and encapsulated siRNA/PEI complexes. The encapsulated siRNA/PEI formed at N:P = 6 achieved significantly higher levels of silencing compared to the unencapsulated complexes at N:P = 6 and three other targeted PEGylated liposome siRNA‐encapsulated formulations. HPV‐E7 mRNA expression was reduced to 32 ± 7% of control levels with the N:P = 6 encapsulated siRNA/PEI complexes, a 10‐fold improvement in silencing over free siRNA delivery (unencapsulated and uncomplexed siRNA, N:P = 0). mRNA expression (106 ± 9% compared to untreated cells) was unaffected by delivery of targeted PEGylated liposomes encapsulating a non‐silencing control siRNA complexed with PEI at N:P = 6, indicating that targeted liposomes delivering control siRNA do not induce mRNA silencing. Notably, the encapsulated siRNA/PEI complexes at N:P = 6 achieved a 2.6‐fold decrease in expression compared to targeted PEGylated liposomes encapsulating uncomplexed siRNA (N:P = 0). Since the same siRNA concentration was used for all formulations, this suggests a benefit to transfection from the presence of PEI. The silencing achieved by the highest performing targeted PEGylated liposome formulation (complexed siRNA/PEI at N:P = 6 encapsulated in targeted PEGylated liposomes) was also compared to the silencing efficiency from non‐targeted liposomes encapsulating siRNA/PEI complexes at the same N:P ratio and from Lipofectamine RNAimax, a commercial RNAi transfection reagent (Figure 7). The siRNA/PEI encapsulated in the targeted PEGylated liposomes achieved 1.9‐fold decreased expression compared to the non‐targeted formulations, and 2.2‐fold decrease in expression compared to Lipofectamine, displaying superior *in vitro* delivery and transfection ability.

### 2.6 Internalization of siRNA encapsulated in targeted PEGylated liposomes

**Figure 8:**
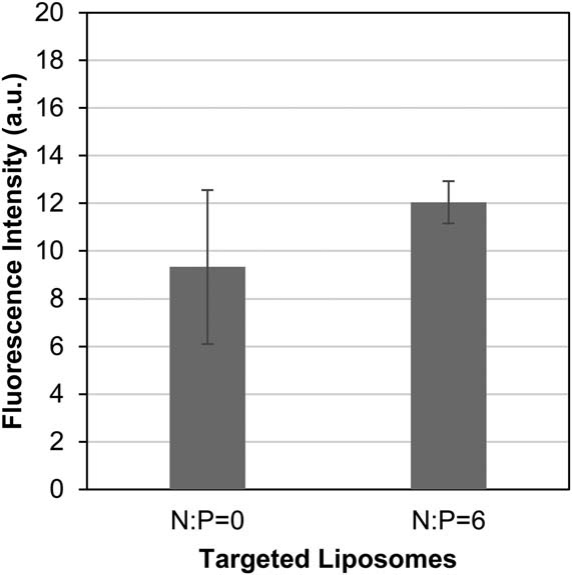
Binding and internalization of targeted PEGylated liposomes encapsulating complexed siRNA/PEI (N:P = 6, 5.5 ± 0.2 mol% AG86) or uncomplexed siRNA (N:P = 0, 5.8 ± 0.2 mol% AG86) quantified using flow cytometry. 2.5 nM of Cy5‐labelled siRNA were delivered to HeLa cells for 24 hr. Background fluorescence of untreated cells was subtracted from data. Data are normalized to fluorescence vs. concentration standards for each liposome batch. Data are presented as the mean ± SE (*n* = 6). There was no significant statistical difference between the pair of means

In order to investigate further the benefit observed from complexation of siRNA with PEI before encapsulation, targeted PEGylated liposomes encapsulating uncomplexed siRNA (N:P = 0) or siRNA/PEI complexes (N:P = 6) were delivered to HeLa cells and the fluorescence intensity from the Cy5‐labeled siRNA was used to compare the amount of the fluorescently labeled siRNA in the cells through binding and internalization of the two formulations. The fluorescence intensity from delivery of the targeted liposomes encapsulating complexed or uncomplexed siRNA was similar (Figure 8). The effect of siRNA loading in the targeted PEGylated liposomes was also considered. The theoretical loaded fraction was calculated as reported previously,^27^ using an average of 26.8 siRNA molecules per siRNA/PEI complex based on the number of siRNA‐PEI binding sites (*n* = 0.57 ± 0.02) calculated using the ITC data shown in Figure 4. The theoretical loading of the uncomplexed siRNA in the targeted PEGylated liposomes is 100% compared to 34.0 ± 3.9% for the complexed siRNA. A difference in the fraction of loaded liposomes would therefore not explain the difference in transfection efficiency and since the presence of PEI has no apparent effect on the binding and internalization efficiency of the targeted liposomes encapsulating the siRNA, this suggests that the improved transfection efficiency observed in the presence of PEI is realized after internalization.

### 2.7 Cytotoxicity of siRNA encapsulated in targeted PEGylated liposomes

**Figure 9:**
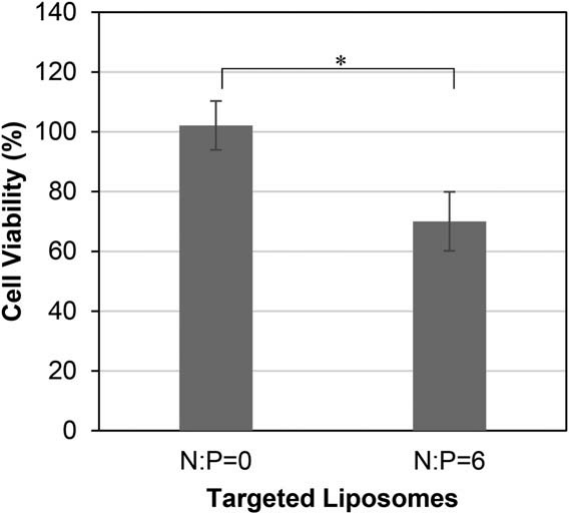
Cytotoxicity of targeted PEGylated liposomes encapsulating siRNA either complexed with PEI (siRNA/PEI at N:P = 6, 5.5 ± 0.2 mol% AG86) or uncomplexed siRNA (N:P = 0, 5.8 ± 0.2 mol% AG86). 2.5 nM of siRNA were delivered to HeLa cells for 24 hr and toxicity from HPV‐E7 silencing was measured by comparing cell viability of treated and untreated cells. Data are presented as the mean ± SE (*n* = 4, performed in triplicate). * *p* < 0.05

The optimized targeted PEGylated liposomes encapsulating siRNA/PEI at N:P = 6 were evaluated further for their cytotoxicity against α_6_β_4_‐expressing, HPV‐18‐positive HeLa cervical cancer cells *in vitro*. The targeted PEGylated liposomes encapsulating either complexed (N:P = 6) or uncomplexed siRNA (N:P = 0) were delivered to HeLa cells and cell viability was assayed 24 hr later. As Figure 9 shows, the nanoparticles with the complexed siRNA decreased cell proliferation by 30%, while those encapsulating the uncomplexed siRNA had no effect on cell proliferation. 2.5 nM siRNA delivered using targeted PEGylated liposomes achieved 68% mRNA silencing, as shown in Figure 6, however protein expression and subsequent phenotypic effects are not quantitatively predicted by mRNA expression and may account for the observed 30% cell toxicity.^43^, ^44^ Our results are in agreement with previous findings where it was shown that silencing of the HPV‐E7 gene using RNAi promoted 80–90% mRNA silencing and resulted in 40–60% inhibition of cell proliferation at early time points.^12^, ^45^ Therefore, continued doses of siRNA may be necessary to achieve more potent cytotoxicity effects. The cytotoxicity from the individual components of the targeted PEGylated liposomes encapsulating siRNA/PEI was also investigated. Empty targeted PEGylated liposomes and siRNA/PEI complexes of a non‐specific sequence were delivered to the HeLa cells either free in solution or encapsulated in the targeted liposomes. As Supporting Information Figure S4 shows, none of the isolated components exhibited significant cytotoxicity compared to the untreated control, thus concluding that the toxicity observed in Figure 9 was the result of transfection of the si18E7‐674 siRNA sequence.

### 2.8 Cell apoptosis induced by different formulations

**Figure 10:**
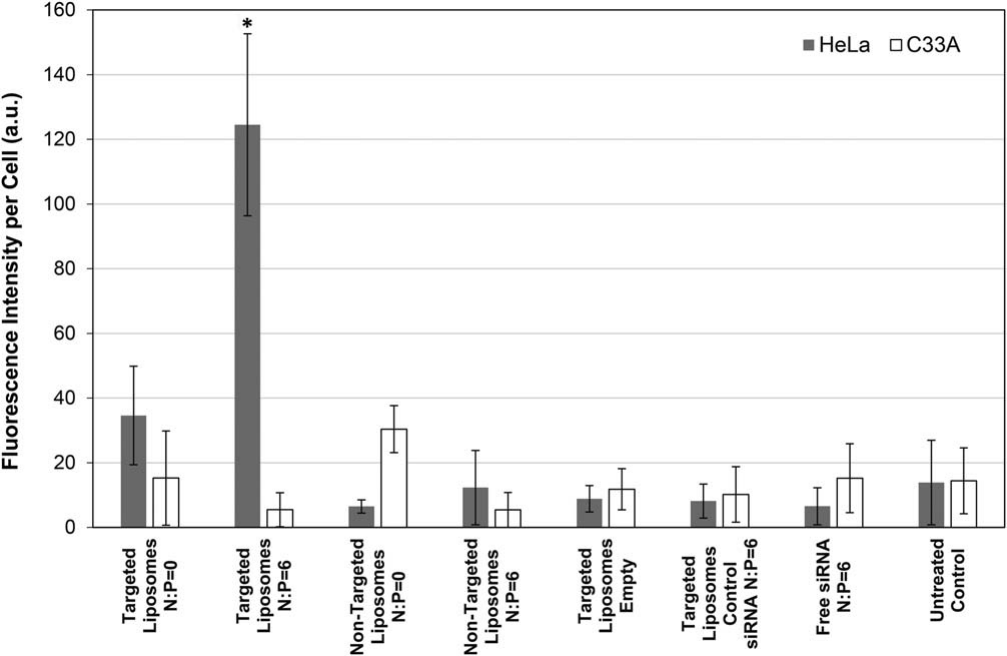
Apoptosis of HPV‐18‐positive HeLa cervical cancer cells and HPV‐negative C33A cervical cancer cells after incubation for 24 hr with 2.5 nM of complexed siRNA/PEI (N:P = 6) encapsulated in targeted and non‐targeted liposomes or delivered free in solution. 2.5 nM of uncomplexed siRNA (N:P = 0) was also delivered to cells encapsulated in targeted and non‐targeted liposomes. Other controls included empty targeted liposomes (750 nM lipids), targeted liposomes encapsulating 2.5 nM of a control siRNA complexed with PEI at N:P = 6 and untreated cells. All liposomes were PEGylated and the targeted liposomes were prepared with 5 mol% AG86. Data are presented as the mean ± SE (*n* = 3, performed in quintuplicate). The targeted liposomes encapsulating siRNA/PEI (N:P = 6) were the only formulation that was statistically different from all other samples, and only when delivered to HeLa cells (* *p* < 0.01)

The oncogenic behavior exhibited by the E7 viral oncogene results from its ability to bind pRb, a tumor suppressor protein that participates in apoptosis and cell cycle regulation.^8^, ^12^, ^46^ An apoptosis assay was therefore performed to investigate the role of apoptosis in the cytotoxicity observed from delivery of E7‐specific siRNA encapsulated within targeted PEGylated liposomes as shown in Figure 9. siRNA was delivered to the HPV‐18‐positive HeLa cells either complexed with PEI (N:P = 6) or uncomplexed (N:P = 0) and encapsulated in both targeted and non‐targeted PEGylated liposomes in order to further evaluate the targeting ability of the AG86 ligand. Complexed siRNA was also delivered free in solution. These formulations were compared to targeted PEGylated liposomes that were either empty or were encapsulating a control siRNA complexed with PEI (N:P = 6) to evaluate the toxicity of the delivery vehicle components. To further evaluate the targeting specificity of the siRNA sequence, all formulations were delivered to HPV‐negative C33A cervical cancer cells. A characteristic feature of apoptosis is the exposure of the lipid phosphatidylserine (PS) to the outer cell membrane that is confined to the inner membrane in healthy cells. Thus, cell apoptosis was detected in this study using fluorescently labeled annexin‐V, a PS‐binding protein. Propidium iodide (PI), a membrane‐impermeable DNA dye, was also used to stain necrotic cells. The disadvantage of this assay is that necrotic cells are labeled on rupture of their plasma membrane. In this study, PI‐positive necrotic cells were not detected and it was hypothesized that it was due to their removal during the washing step required by the assay. Figure 10 shows the fluorescence detected from the annexin‐V‐positive apoptotic cells scaled by the number of cells in each image. Results showed that the only formulation that causes statistically significant apoptosis of the HPV‐18‐positive HeLa cells while leaving the HPV‐negative C33A cells unaffected, was the complexed siRNA (N:P = 6) encapsulated in the targeted PEGylated liposomes. These data thus validate the cell viability results of Figure 9 and Supporting Information Figure S4. The results also highlight our approach of using AG86‐functionalized liposomes that bind to α_6_β_4_‐expressing HeLa cells, along with the si18E7‐674 that is shown to be specific for the HPV‐18 containing HeLa cells, as the formulation had no effect on the HPV‐negative C33A cells. Furthermore, the lack of apoptotic signal from the delivery of targeted liposomes that were either empty or loaded with a complexed control siRNA demonstrates the safety of our delivery system itself.

## 3 Discussion

Numerous gene silencing targets with therapeutic potential to treat cancer, viral infections and respiratory diseases have been identified, but effective systemic gene delivery strategies are critical for successful pursuit of these genetic targets.^3^, ^4^, ^13^ While cationic polyplexes or lipoplexes mediate extremely effective RNAi transfection *in vitro*, their instability and immunogenicity *in vivo* preclude their clinical success, which has inspired numerous synthetic alternatives.^2^, ^5^, ^17^, ^42^, ^47^, ^48^ Most notably, stable nucleic acid lipid particles (SNALPs), liposome‐polycation‐DNA, cyclodextran nanoparticles, and lipidoids are cationic lipid and polymer based nanoparticles that have become extremely popular for preclinical exploration of siRNA delivery for cancer therapy.^1^, ^2^, ^49^ Liposome‐polycation‐DNA particles composed of both DNA and siRNA complexed with protamine, coated with cationic liposomes, shielded with PEG and targeted to a lung cancer biomarker were used to deliver growth receptor silencing siRNA to lung tumors in mice and achieved extremely high tumor localization. Interestingly, the presence of the targeting ligand did not improve tumor localization, but did mediate enhanced cellular uptake and therefore silencing and tumor cell apoptosis. However, the targeted nanoparticles also elicited high levels of cytokine production.^50^ siRNA complexed with a cationic peptide, encapsulated in cationic liposomes and coated in cleavable PEG were designed for the PEG to be cleaved on exposure to tumor associated matrix metalloproteinases. The addition of PEG or cleavable PEG improved tumor accumulation 10–20‐fold over naked nanoparticles, but silencing was significantly improved only in nanoparticles modified with cleavable PEG, allowing for internalization and transfection.^51^ A large lipidoid library was synthesized to develop structure function relationships for amine functionalized lipid molecules used for the production of biodegradable siRNA lipidoid nanoparticles.^52^ Structure function relationships developed from transfection efficiency analysis of this lipidoid library were used to predict and design highly efficient siRNA delivery vehicles. The optimal PEGylated siRNA lipidoid nanoparticles achieved more than 95% protein silencing *in vivo* in hepatocytes.^52^ Interestingly, surface pKa of a particular lipidoid was a critical parameter for predicting transfection efficiency for a lipidoid nanoparticle.^52^ The SNALPs are a well‐studied non‐targeted lipid based delivery vehicle which has seen great success in preclinical studies. In one study, SNALPs were used to silence polo‐like kinase 1 in tumors while abrogating activation of innate immune response and reducing tumor size by 75%.^53^ Transferrin targeted cyclodextrin particles have also successfully delivered siRNA to tumors *in vivo*, and an accumulation/function study again revealed similar tissue accumulation for targeted and non‐targeted nanoparticles, but enhanced transfection associated with targeting.^54^ Preclinical results from SNALP and cyclodextran nanoparticle development motivated clinical trials for oncogene silencing and cancer treatment.^49^ With these advances, many biodistribution challenges have been addressed, and often the key barrier lies in specific cellular uptake and appropriate intracellular release.^13^, ^55^

Previous work within our group has shown that pDNA condensed with PEI can be fully encapsulated within neutral PEGylated liposomes composed of the same materials as FDA approved doxil and other clinical liposomal cancer treatments.^27^, ^56^ PEGylated liposomes encapsulating pDNA/PEI complexes were functionalized with the PR_b targeting peptide for specific delivery to α_5_β_1_‐expressing cancer cells and demonstrated excellent *in vivo* delivery and transfection in a metastatic colorectal mouse model.^56^ Furthermore, a modular multifunctional gene delivery system was prepared in our group by combining the extracellular targeting ability of the PR_b‐functionalized PEGylated liposomes with a new form of transcriptional targeting, by designing a therapeutic pDNA under the control of an NF‐κB responsive element. The targeted nanoparticles (encapsulating pDNA/PEI complexes) specifically killed close to 70% of cancer cells while minimally affecting healthy cells *in vitro*.^27^ Mechanistic investigations revealed that PEGylated liposomes targeting the α_5_β_1_ integrin and encapsulating pDNA/PEI complexes internalized into DLD‐1 colorectal cancer cells through macropinocytosis and caveolar mediated endocytosis. The targeted PEGylated liposomes achieved higher transfection efficiency and more efficient endosomal release than pDNA/PEI complexes alone, suggesting a cooperative effect on internalization and intracellular delivery mediated by the targeted PEGylated liposomes encapsulating pDNA/PEI complexes.^29^

Our *in vitro* and *in vivo* delivery success with plasmid transfection prompted translation of targeted PEGylated liposomes encapsulating pDNA condensed with PEI to the equally challenging technology of specifically delivering siRNA. Our discoveries, along with evidence in the literature from various nonviral gene delivery vehicles that the key delivery barriers may lie in intracellular activity,^13^, ^27^ inspired the hypothesis that PEI complexation of siRNA, though not necessary for encapsulant size considerations as in the case of pDNA, may provide additional benefits to siRNA delivery through targeted PEGylated liposomes. In this work, we designed AG86‐functionalized PEGylated liposomes that targeted α_6_β_4_‐expressing HeLa cervical cancer cells with the goal of delivering the si18E7‐674 siRNA sequence, developed to silence the HPV‐18 E7 gene. The delivery vehicle design considerations addressed here included identifying 5 mol% as a sufficient AG86 peptide concentration for binding and internalization, exploring the necessity of PEI complexation before encapsulation, and then identifying the optimal N:P ratio for effective transfection efficiency.

ITC analysis demonstrated that siRNA complexes are saturated with PEI at N:P = 2, however, increasing the N:P ratio increased the zeta potential of the complexes. This seemingly conflicting result seems to support the binding model proposed in the literature, where siRNA escapes from initial nanocomplexes near N:P = 1 and reorganizes into less siRNA dense nanoparticles in the presence of additional polymer,^39^, ^57^, ^58^ which would result in more positive charge dense complexes without significantly changing complex size. siRNA complexed with PEI was efficiently encapsulated in targeted PEGylated liposomes, achieving on average 60% encapsulation yield, where other neutral lipid based vehicles encapsulated 10% or less.^42^ It was found that there was no significant statistical difference in encapsulation yield between targeted PEGylated liposomes encapsulating siRNA/PEI complexed at different N:P ratios, while silencing increased with increasing N:P up to N:P = 6. Others have also observed an increase in siRNA transfection using PEI with increasing N:P up to a certain ratio, followed by a decrease in transfection efficiency. The increase in transfection with increasing N:P ratio was attributed to higher uptake, and the decrease attributed to increasing cytotoxicity at higher N:P ratios.^24^, ^33^, ^36^, ^39^ Since binding and internalization of the targeted PEGylated liposomes is largely driven by the targeting peptide as shown before and in Figure 2,^29^ and not by the presence of PEI in the liposomes, this explanation is insufficient in our case. Therefore, we speculated that the increase in siRNA silencing with an optimal N:P ratio of the encapsulated siRNA/PEI was more likely caused by an optimization of the local buffering capacity within the liposome.^59^, ^60^, ^61^ Since targeted PEGylated liposomes encapsulating siRNA/PEI complexes at N:P = 6 showed no difference in binding and internalization compared to liposomes encapsulating uncomplexed siRNA (N:P = 0), as shown in Figure 8, but achieved 3.7‐fold higher silencing efficiency (Figure 6), this indicates that the PEI complexation of siRNA in these gene delivery vehicles may have improved efficiency either through carrier release, endosomal escape, or a combination of both. PEI mediated endosomal release could minimize immunogenicity, as it has been found that the endosomal acidification process is crucial to siRNA induction of the interferon and cytokine response.^62^

Active targeting has been shown to significantly improve transfection for several other gene delivery vehicles.^50^, ^54^, ^63^ Targeting can be desirable as it can both increase the concentration of internalized siRNA and influence trafficking pathways into the cell. Therefore, in addition to any benefit PEI may provide, the actual internalization pathway of the targeted delivery vehicle after binding to the receptor of choice may play a synergistic role, as shown in the case of PEGylated liposomes targeting the α_5_β_1_ integrin and encapsulating pDNA/PEI complexes.^29^ The internalization pathways of the α_6_β_4_ integrin have yet to be elucidated,^32^, ^64^, ^65^, ^66^, ^67^ but it is possible that they may play a synergistic role in transfection efficiency. Targeted PEGylated liposomes encapsulating siRNA/PEI at N:P = 6 decreased the proliferation of HeLa cells by 30% on average at 2.5 nM si18E7‐674, and induced increased cell apoptosis specifically to the HPV‐18‐positive HeLa cells thus demonstrating a potential therapeutic effect for this system while suggesting that repeated doses of this siRNA sequence may be necessary to achieve more potent cytotoxicity effects. The apoptosis results shown in Figure 10 reiterate the conclusion that both the targeting and the PEI condensation are critical for effective delivery of the HPV‐E7 siRNA, as significant apoptosis occurred in HeLa cells only with delivery of targeted PEGylated liposomes encapsulating siRNA/PEI complexed at N:P = 6. Much lower levels of apoptosis were observed from siRNA delivery without targeting, without PEI complexation (N:P = 0), and without both. In addition, si18E7‐674 delivered within α_6_β_4_ integrin‐targeted liposomes demonstrated specificity of apoptosis induction for the disease state cell line (HeLa) containing the HPV‐18 genome and expressing higher levels of the α_6_β_4_ integrin, compared to the control cell line (C33A) which does not contain an HPV genome and expresses lower levels of the α_6_β_4_ integrin.^68^ Cancer gene therapy using a viral‐specific siRNA sequence delivered within a cancer biomarker‐targeted vehicle takes advantage of two levels of targeting, requiring the presence of both the targeted biomarker and the targeted viral gene. Delivery schemes utilizing multiple levels of targeting create therapies that are more selective and therefore more effective for treating cancer.

## 4 Conclusions

A significant challenge for cancer therapy lies in the effective discrimination between healthy and tumor tissue. Targeting delivery vehicles to cancer biomarkers in order to improve delivery specificity has been explored in depth, however, off target delivery is a common limitation of this scheme.^69^, ^70^ Additional genetically mediated targeting could potentially overcome this obstacle by removing off target effects from the therapy itself.^28^, ^71^, ^72^ Each aspect of the targeted PEGylated liposome encapsulating siRNA/PEI complexes, provided a significant delivery mediator: the vehicle targeting mediating cell internalization and improving bioavailability of the therapy at the tumor site, and the viral‐specific gene silencing minimizing off target effects since healthy cells lack the targeted viral genome. Combined with the improvement to intracellular availability and transfection through PEI complexation, targeted PEGylated liposomes encapsulating siRNA/PEI complexes provide an example of a modular gene delivery vehicle designed to address each barrier of gene therapy.

## 5 Materials and methods

### 5.1 siRNA labeling and quantification

si18E7‐674 (CTAGCACGAGCAATTAAGCGA), shown to silence the E7 oncogene of HPV‐18 in HPV‐18 containing cells,^12^ (GE Dharmacon, Lafayette, CO) and used throughout this study, was fluorescently labeled using Cy5 Label IT Tracker Intracellular Nucleic Acid Localization Kit (Mirus, Madison, WI). The conjugation was carried out according to the manufacturer's protocol using the maximum recommended reagent volume and a reaction time of 3 hr. For liposome encapsulated siRNA used for silencing, 5% of the total siRNA encapsulated was labeled with Cy5. For liposome encapsulated siRNA used for siRNA binding and internalization studies, 25% of the total siRNA encapsulated was labeled with Cy5. An siRNA concentration standard curve was produced from the complexed siRNA following the hydration step of liposome formation.^27^ Fluorescence intensity of the standard and of the liposome sample were measured using a Synergy H1 fluorescence microplate reader (Biotek, Winooski, VT). Yield was calculated as final siRNA content after purification compared to initial siRNA content used for encapsulation (100 pmol).

### 5.2 siRNA/PEI complexation

100 pmol of total siRNA (5% fluorescently labeled siRNA, si18E7‐674^1^ 2) was complexed using 25 kDa branched PEI (Sigma‐Aldrich, St. Louis, MO). 100 pmol of siRNA was dissolved in 6 mM HEPES buffer, mixed with an equal volume of PEI in 6 mM HEPES at the desired amine to phosphate (N:P) ratio and a final siRNA concentration of 67 nM, was vortexed for 5 s and incubated at room temperature for 20 min. The size of complexed siRNA nanoparticles was measured using a NanoSight LM10 (Malvern Instruments, Malvern, UK) with a 405 nm laser, and zeta potential analysis (Brookhaven Instruments Corporation, Holtsville, NY) was used to measure the surface charge of the particles.

### 5.3 Isothermal calorimetry (ITC)

siRNA and PEI were prepared in the same buffer to minimize mixing effects. PEI was then injected at 2 μL increments into the siRNA solution using MicroCal Auto‐iTC 200 (Malvern Instruments, Malvern, UK) to measure the power required to maintain a constant chamber temperature. Using the MicroCal Auto‐iTC200 software, the resulting power versus time data were integrated to determine heat exchange associated with each injection. The heat of dilution was accounted for by subtracting the heat data obtained from injections of PEI into buffer from the heat data obtained from injecting PEI into an siRNA solution. The resulting heat curve was fitted to a “one set of sites” binding model, which uses *n*, the number of binding sites, *K*, the binding association constant, and *ΔH*, the enthalpy of binding, as fitting parameters where all *n* binding sites have the same *K* and *ΔH*.^38^, ^40^ The data were fitted to the following equations:

(1) $$K=\;\frac{\theta }{\left(1-\theta \right)[X]}$$

(2) $$X_{t}=\left[X\right]+\;n\theta M_{t}$$

(3) $$Q=n\theta M_{t}\Delta HV_{o}$$

(4) $$\Delta Q\left(i\right)=\;Q\left(i\right)+\frac{dV_{i}}{V_{o}}\left[\frac{Q\left(i\right)+\;Q\left(i-1\right)}{2}\right]-Q\left(i-1\right)$$

where  θ is the fraction of sites occupied by the siRNA, $\left[X\right]$ is the free siRNA concentration, $X_{t}$ is the bulk siRNA concentration, $M_{t}$ is the bulk PEI concentration, *Q* is the total heat content of the solution, $\Delta Q\left(i\right)$ is the heat released from the *i*th injection, $\;V_{o}$ is the volume of the chamber, and $dV_{i}$ is the injection volume at the *i*th injection.

### 5.4 Liposome formation

1,2 Dipalmitoyl‐sn‐glycero‐3‐phosphocholine (DPPC), 1,2‐dipalmitoyl‐sn‐glycero‐3‐phosphoethanolamine‐N‐[methoxy(poly(ethylene glycol))‐2000] (ammonium salt) (PEG2000), and cholesterol were purchased from Avanti Polar Lipids, Inc. (Alabaster, AL). AG86 peptide (KSSLGGLPSHYRARNI)^23^ was purchased from United Biosystems (Herndon, VA) and the C_16_ double tail peptide‐amphiphile was synthesized as described previously.^69^ *x* mol% AG86 peptide‐amphiphile (*x* = 0–10), (60‐x) mol% DPPC, 35 mol% cholesterol and 5 mol% PEG2000 dissolved in chloroform were combined in a round bottom flask at a total lipid content of 5 μmol and dried under a stream of argon. The solution was dried under argon at 60 °C to produce a homogeneous lipid film, followed by overnight incubation in a vacuum oven. This film was hydrated with 0.75 ml of 2 mM calcein or 1.5 ml of complexed siRNA/PEI at 45 °C for 1.5 hr. These liposomes were extruded 11 times using a manual extruder (Avestin, Ottowa, ON) through 200 nm membranes. For siRNA liposomes, the unencapsulated siRNA was removed with overnight dialysis purification through a 1000 kDa MWCO membrane (Spectrum Laboratories, Rancho Dominguez, CA) and stored at 4–8 °C for up to 4 weeks.^27^, ^56^ Calcein liposomes were purified using a Sepharose CL‐4B gel filtration column to remove unencapsulated material and stored at 4–8 °C for up to 4 weeks. Lipid concentration was measured using a phosphorous assay as described elsewhere^73^ and peptide concentration was measured using a BCA Protein Assay Kit (Pierce Biotechnology, Rockford, IL). Liposome size and charge were measured using dynamic light scattering (DLS) and zeta potential analysis (Brookhaven Instruments Corporation, Holtsville, NY). A fluorescence standard relating calcein fluorescence to total lipid concentration was used to quantify the amount of bound and internalized lipids for calcein liposome experiments. siRNA quantification is described above.

### 5.5 Binding and internalization of fluorescent liposomes

Human cervical adenocarcinoma HeLa cells (ATCC, Manassas, VA) were subcultured in black 96‐well plates at 5,000 cells/well containing 200 μL Minimum Essential Media (MEM) supplemented with 10% fetal bovine serum (FBS), 100 units/ml penicillin and 0.1 mg/ml streptomycin. Targeted PEGylated liposomes encapsulating 2 mM calcein with varying AG86 content (0–10 mol%) were incubated with HeLa cells for 3, 6, and 24 hr at 37 °C and 5% CO_2_ at a lipid concentration of 100 μM. After incubation, the cells were washed 3 times with phosphate buffered saline (PBS) and maintained at −80 °C for 24 hr. The frozen cells were then thawed, lysed with lysis buffer (Promega, Madison, WI) and the calcein fluorescence was quantified at excitation/emission wavelengths of 485/515 nm. The total amount of bound and internalized lipids was calculated based on a standard curve relating fluorescence and lipid concentration.

### 5.6 Peptide and antibody blocking of fluorescent liposome binding

HeLa cells were fixed by incubation in 4% paraformaldehyde (Sigma, St. Louis, MO) for 10 min at 25 °C, and aliquoted in 4 °C 1% w/v bovine serum albumin (ThermoFisher Scientific, Grand Island, NY), 0.9 mM CaCl_2_, 0.5 mM MgCl_2_ PBS at 5 × 10^6^ cells/ml. Cells were incubated with 10 µg/ml AG86 peptide, 1:100 dilution of rat anti‐human CD49f/α_6_ (eBioscience, Inc., San Diego, CA), or 1:100 dilution of MAΒ1964 mouse anti‐human β_4_ (EMD Millipore, Darmstadt, Germany) at 4 °C for 30 min. AG86‐functionalized PEGylated liposomes (50 µM lipid concentration) encapsulating 2 mM calcein were added and incubated at 4 °C for 1 hr, then washed twice with 4 °C PBS and analyzed immediately on a BD Accuri C6 flow cytometer (Masonic Cancer Center, University of Minnesota).

### 5.7 Silencing of HPV‐E7 gene mRNA

HeLa cells were subcultured in clear 12‐well plates at 50,000 cells/well in 2 ml of MEM supplemented with 10% FBS, 100 units/ml penicillin and 0.1 mg/ml streptomycin and incubated for 24 hr at 37 °C and 5% CO_2_. Media was replaced and 2.5 nM si18E7‐674^1^ 2 was delivered to each well with the different transfection agents for 24 hr at 37 °C and 5% CO_2_. RNA extraction was conducted using E.Z.N.A Total RNA Isolation Kit I (Omega Biotek, Norcross, GA) according to the manufacturer's protocol. The concentration of extracted RNA was quantified using an absorbance microspot reader (Biotek, Winooski, VT) and 1 μmol of RNA was converted to cDNA using RNA to cDNA EcoDry™ Premix (Double Primed) according to the manufacturer's protocol (Clontech, Mountain View, CA). HPV‐E7 expression was quantified through a real‐time reverse transcription polymerase chain reaction (qRT‐PCR) using PerfeCTa qPCR mix (Quanta Biosciences, Gaithersburg, MD) in a MX3000P qPCR machine (Agilent, Santa Clara, CA). The geometric means of the reference gene threshold cycles (*C_t,ref1_*, *C_t,ref2_*) were used to normalize the HPV‐E7 threshold cycle for each sample. TATA‐binding protein (TBP) and tyrosine 3‐monooxygenase activation protein‐zeta (YWHAZ) were chosen as reference genes to minimize the effect of apoptosis on reference gene expression and subsequent mRNA expression measurements.^74^ A buffer and RNA control were included for each experiment. The reference gene threshold was calculated as

(5) $$C_{t,ref}=\sqrt{C_{t,ref1}xC_{t,ref2}}$$

where *C_t,ref1_* and *C_t,ref2_* are the threshold cycles of the individual reference genes.

HPV‐E7 gene silencing was calculated using the following equations. First, the normalized sample threshold cycle, Δ*C_t_*, was determined by subtracting the reference gene threshold cycle (*C_t,ref_*) from the target gene threshold cycle (*C_t,x_*) for each sample:

(6) $$\Delta C_{t}=C_{t,x}-C_{t,ref}$$

The difference between treated and untreated threshold cycles, ΔΔ*C_t_*, was then calculated by subtracting the Δ*C_t,untreated_* of the untreated sample from the Δ*C_t,treated_* of the treated sample:

(7) $$-\Delta \Delta C_{t}=-(\Delta C_{t,treated}-\Delta C_{t,untreated})$$

The fold silencing that was achieved in the treated sample relative to the untreated sample was calculated as follows:

(8) $$Fold\;Decrease=(2^{-\Delta \Delta C_{t}})$$

### 5.8 Binding and internalization of siRNA liposomes

HeLa cells were subcultured in clear 12‐well plates at 50,000 cells/well in 2 ml of MEM supplemented with 10% FBS, 100 units/ml penicillin and 0.1 mg/ml streptomycin and incubated for 24 hr at 37 °C and 5% CO_2_. Media was replaced and 2.5 nM encapsulated si18E7‐674 was delivered and allowed to incubate for 24 hr at 37 °C and 5% CO_2_. Cells were then harvested with TrypleE Express cell dissociation agent (ThermoFisher Scientific, Grand Island, NY), pelleted by centrifugation at 500 *g* for 2.5 min, washed twice with 4 °C PBS and analyzed immediately on a BD Accuri C6 flow cytometer (Masonic Cancer Center, University of Minnesota). Fluorescence intensities were normalized to siRNA concentration with the concentration standard used to determine yield.

### 5.9 Cell viability

HeLa cells were subcultured in clear 96‐well plates at 5,000 cells/well in 200 µL of MEM supplemented with 10% FBS, 100 units/ml penicillin and 0.1 mg/ml streptomycin and incubated for 24 hr at 37 °C and 5% CO_2_. Media was replaced and 2.5 nM liposome encapsulated si18E7‐674 or non‐specific control siRNA (siGENOME Non‐Targeting siRNA #2, GE Dharmacon, Lafayette, CO) or empty liposomes (750 nM lipids) were delivered and allowed to incubate for 24 hr at 37 °C and 5% CO_2_. Cell viability was then measured using a WST‐1 Cell Proliferation Reagent (Roche, Indianapolis, IN) following the manufacturer's protocol. Absorbance was measured using a Synergy H1 microplate reader (Biotek, Winooski, VT). Cell viability was normalized to untreated cells.

### 5.10 Cell apoptosis

HPV‐negative human cervical carcinoma C33A cells (ATCC, Manassas, VA) and HPV‐18‐positive HeLa cells were subcultured at 30,000 cells/well on fibronectin‐coated 20 mm glass coverslips (Neuvitro, Vancouver, WA) placed into clear 12‐well plates in 1 ml of Dulbecco's Modified Eagle Medium (DMEM) (ThermoFisher Scientific, Waltham, MA) supplemented with 10% FBS, 100 units/ml penicillin and 0.1 mg/ml streptomycin and incubated for 24 hr at 37 °C and 5% CO_2_. Media was replaced and 2.5 nM siRNA (si18E7‐674 or control) was added, either encapsulated in liposomes or free in solution, and allowed to incubate for 24 hr at 37 °C and 5% CO_2_. 750 nM lipid concentration was added for the empty liposomes. The apoptosis assay was performed using the Dead Cell Apoptosis Kit with Annexin V Alexa Fluor 488 & propidium iodide (ThermoFisher Scientific, Waltham, MA) according to the manufacturer's protocol. Wells were washed with 500 μL PBS and cells were fixed for 15 min in 4% paraformaldehyde in PBS, followed by a 500 μL PBS wash. Glass coverslips were mounted on ethanol washed Gold Seal Rite‐On Frosted Microslides (ThermoFisher Scientific, Waltham, MA) using ProLong Gold Antifade Mountant (ThermoFisher Scientific, Waltham, MA). The slides were allowed to cure for 24 hr in the dark at room temperature, sealed using clear nail polish and imaged using an EVOS FL microscope (ThermoFisher Scientific, Waltham, MA). Fluorescence was recorded in the green (470/510 nm excitation/emission) and red channels (585/624 nm excitation/emission) and was divided by the number of cells in each image. Images were analyzed using macros written in the Fiji distribution of ImageJ and the number of cells in each image was calculated by thresholding the phase‐contrast channel into a binary (black and white) image and using the built‐in “Analyze Particles” function.

### 5.11 Statistics

ANOVA analysis and Tukey's honest significant difference (HSD) test were performed to calculate *p*‐values and determine statistical significance between means. When only two means were compared within an experiment, student's *t*‐test was used to calculate *p*‐values.

## Supporting information

Additional Supporting Information can be found the online version of this article at the publisher's website.

**Table S1**. *p*‐values from ANOVA statistical analysis for Figure 1 showing binding and internalization of fluorescent liposomes with varying peptide concentration and incubation times at 37 °C.
**Figure S1**. Peptide and antibody blocking of AG86‐functionalized stealth liposomes. 5–6 mol% AG86‐functionalized, calcein loaded, stealth liposomes (green) were delivered at 50 μM lipids to HeLa cells at 4 °C for 1 hr after a 30 min incubation of (A) 10 μg/ml free AG86 peptide (blue) or (B) 100× dilution of anti‐α_6_ (blue) and anti‐β_4_ (red) integrin antibodies. Binding was measured using flow cytometry. Untreated cells (grey) were measured for background fluorescence.
**Figure S2**. Representative histograms of particle size analysis of siRNA/PEI complexes at N:P ratio 2 (A), 4 (B), 6 (C), and 8 (D).
**Figure S3**. Size (A) and zeta potential (B) measurements of targeted stealth liposomes encapsulating siRNA. siRNA/PEI complexes were prepared at various N:P ratios, then encapsulated in stealth liposomes (5–6 mol% AG86) for characterization. N:P = 0 indicates encapsulation of uncomplexed siRNA (no PEI). Empty stealth liposomes (5.3 mol% AG86) were prepared by hydrating lipid films with buffer. Data are presented as the mean ± SE (*n* = 3–6). There was no significant statistical difference for all pairs.
**Figure S4**. Toxicity from the components of targeted stealth liposomes used for siRNA delivery. Empty targeted liposomes (750 nM lipids, 5.3 ± 0.1 mol% AG86) or 2.5 nM siRNA/PEI particles (N:P = 6) of a control non‐silencing siRNA either encapsulated in targeted liposomes (4.9 ± 0.2 mol% AG86) or free in solution were delivered to HeLa cells for 24 hr and toxicity was measured by comparing cell viability of treated and untreated cells. Data are presented as the mean ± SE (*n* = 3, performed in triplicate). There was no significant statistical difference between any pairs.
