# Supplementary material for: Targeting HPV‐infected cervical cancer cells with PEGylated liposomes encapsulating siRNA and the role of siRNA complexation with polyethylenimine
Source: Bioeng Transl Med. 2016 Aug 8;1(2):168–80. doi: 10.1002/btm2.10022 (PMC5675078; doi:10.1002/btm2.10022)
Supplement: Supplementary file 1 — Table S1. p‐values from ANOVA statistical analysis for Figure 1 showing binding and internalization of fluorescent liposomes with varying peptide concentration and incubation times at 37 °C. Figure S1. Peptide and antibody blocking of AG86‐functionalized stealth liposomes. 5–6 mol% AG86‐functionalized, calcein loaded, stealth liposomes (green) were delivered at 50 μM lipids to HeLa cells at 4 °C for 1 hr after a 30 min incubation of (A) 10 μg/ml free AG86 peptide (blue) or (B) 100× dilution of anti‐α6 (blue) and anti‐β4 (red) integrin antibodies. Binding was measured using flow cytometry. Untreated cells (grey) were measured for background fluorescence. Figure S2. Representative histograms of particle size analysis of siRNA/PEI complexes at N:P ratio 2 (A), 4 (B), 6 (C), and 8 (D). Figure S3. Size (A) and zeta potential (B) measurements of targeted stealth liposomes encapsulating siRNA. siRNA/PEI complexes were prepared at various N:P ratios, then encapsulated in stealth liposomes (5–6 mol% AG86) for characterization. N:P = 0 indicates encapsulation of uncomplexed siRNA (no PEI). Empty stealth liposomes (5.3 mol% AG86) were prepared by hydrating lipid films with buffer. Data are presented as the mean ± SE (n = 3–6). There was no significant statistical difference for all pairs. Figure S4. Toxicity from the components of targeted stealth liposomes used for siRNA delivery. Empty targeted liposomes (750 nM lipids, 5.3 ± 0.1 mol% AG86) or 2.5 nM siRNA/PEI particles (N:P = 6) of a control non‐silencing siRNA either encapsulated in targeted liposomes (4.9 ± 0.2 mol% AG86) or free in solution were delivered to HeLa cells for 24 hr and toxicity was measured by comparing cell viability of treated and untreated cells. Data are presented as the mean ± SE (n = 3, performed in triplicate). There was no significant statistical difference between any pairs. [file BTM2-1-168-s001.docx]

**Supplemental Information**

## Targeting HPV-Infected Cervical Cancer Cells with Stealth Liposomes Encapsulating siRNA and the Role of siRNA Complexation with Polyethylenimine

Rachel M. Levine, Christina V. Dinh, Michael A. Harris, Efrosini Kokkoli*

Department of Chemical Engineering and Materials Science,

University of Minnesota, Minneapolis, MN 55455, USA

**Table S1.** p-values from ANOVA statistical analysis for Figure 1 showing binding and internalization of fluorescent liposomes with varying peptide concentration and incubation times at 37 °C.

**A**

**B**

**Figure S1.** Peptide and antibody blocking of AG86-functionalized stealth liposomes. 5-6 mol% AG86-functionalized, calcein loaded, stealth liposomes (green) were delivered at 50 μM lipids to HeLa cells at 4 °C for 1 hr after a 30 min incubation of (A) 10 μg/mL free AG86 peptide (blue) or (B) 100x dilution of anti-α_6_ (blue) and anti-β_4_ (red) integrin antibodies. Binding was measured using flow cytometry. Untreated cells (grey) were measured for background fluorescence.

**
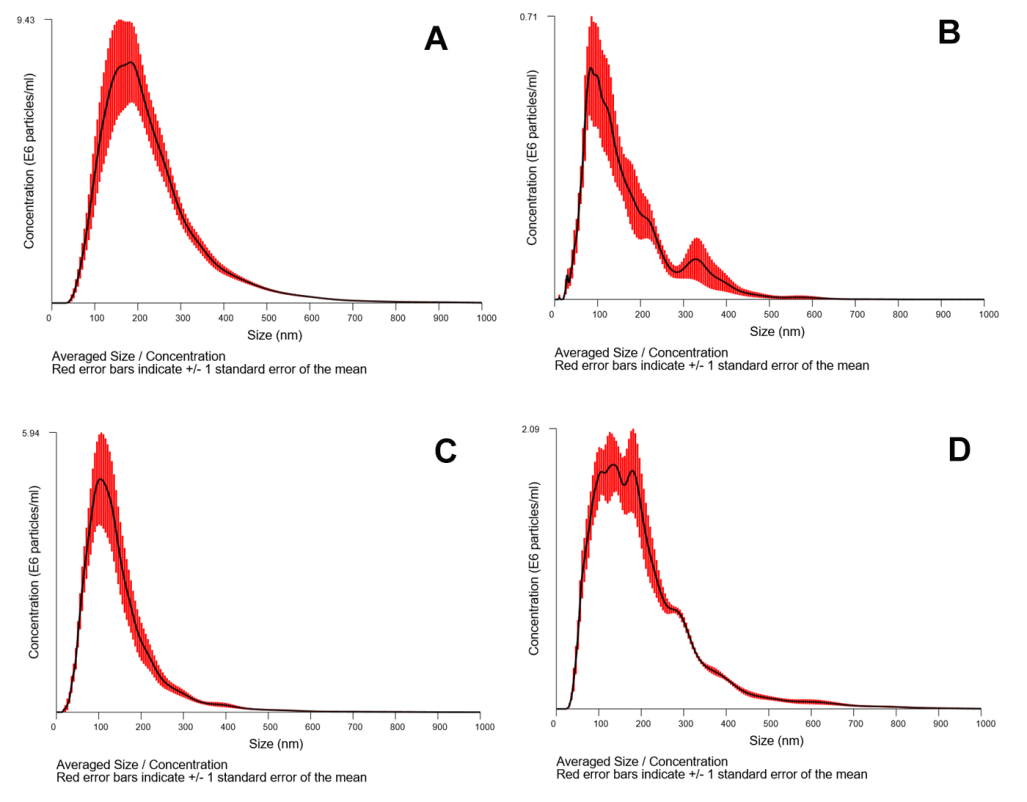
**

**Figure S2.** Representative histograms of particle size analysis of siRNA/PEI complexes at N:P ratio 2 (A), 4 (B), 6 (C) and 8 (D).


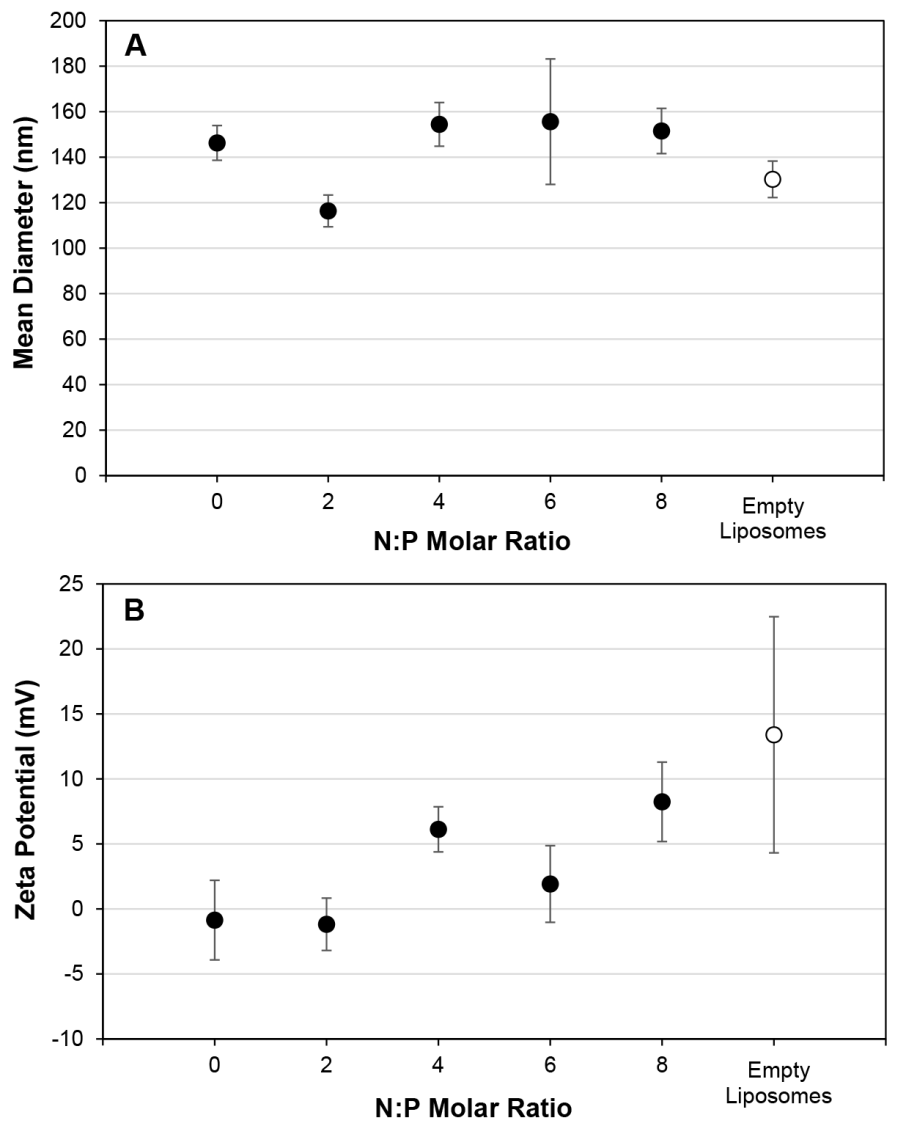


**Figure S3.** Size (A) and zeta potential (B) measurements of targeted stealth liposomes encapsulating siRNA. siRNA/PEI complexes were prepared at various N:P ratios, then encapsulated in stealth liposomes (5-6 mol% AG86) for characterization. N:P = 0 indicates encapsulation of uncomplexed siRNA (no PEI). Empty stealth liposomes (5.3 mol% AG86) were prepared by hydrating lipid films with buffer. Data are presented as the mean ± SE (n = 3-6). There was no significant statistical difference for all pairs.


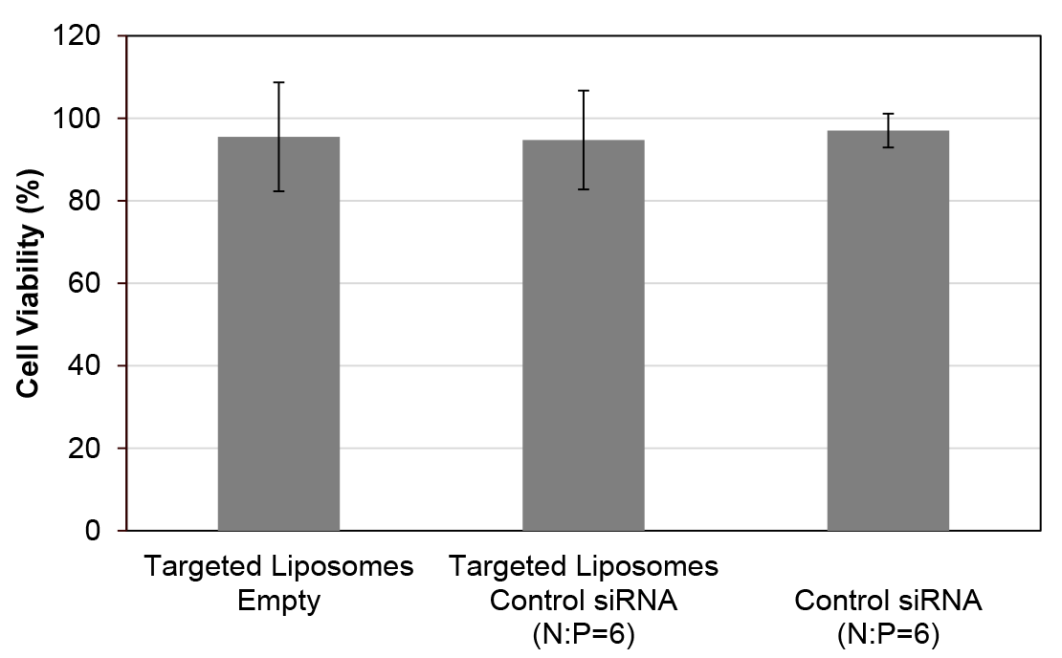


**Figure S4.** Toxicity from the components of targeted stealth liposomes used for siRNA delivery.

Empty targeted liposomes (750 nM lipids, 5.3 ± 0.1 mol% AG86) or 2.5 nM siRNA/PEI particles (N:P = 6) of a control non-silencing siRNA either encapsulated in targeted liposomes (4.9 ± 0.2 mol% AG86) or free in solution were delivered to HeLa cells for 24 h and toxicity was measured by comparing cell viability of treated and untreated cells. Data are presented as the mean ± SE (n = 3, performed in triplicate). There was no significant statistical difference between any pairs.
